# Supplementary material for: Diffusion markers of dendritic density and arborization in gray matter predict differences in intelligence
Source: Nat Commun. 2018 May 15;9:1905. doi: 10.1038/s41467-018-04268-8 (PMC5954098; doi:10.1038/s41467-018-04268-8)
Supplement: Supplementary file 1 — Supplementary Information [file 41467_2018_4268_MOESM1_ESM.pdf]

Diffusion markers of dendritic density and arborization in gray matter predict differences in intelligence

Genç et al.

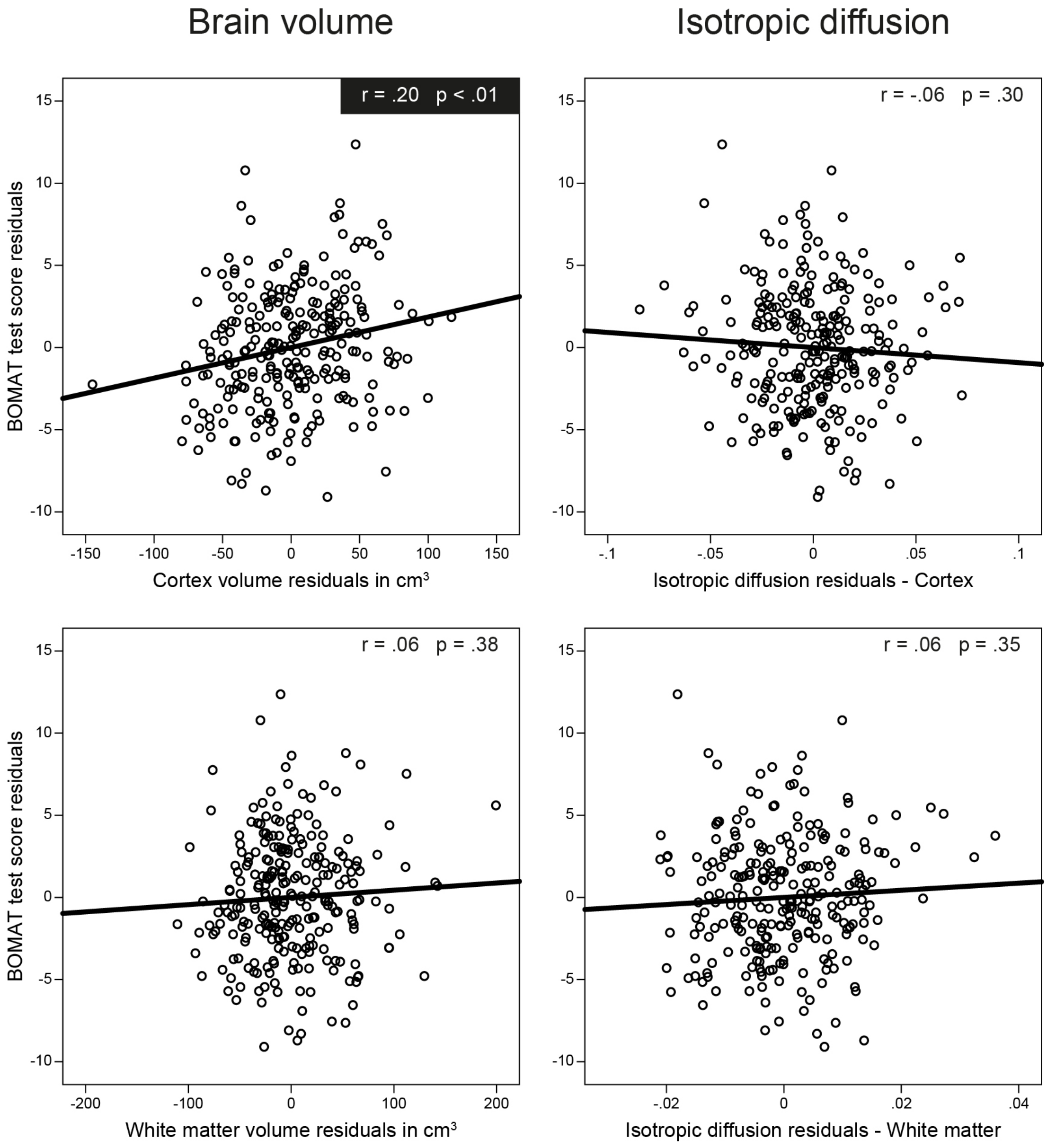

Supplementary Figure 1 - **Partial correlation analyses with data from sample S259 quantifying structure-function associations at the whole-brain level.** Scatter plots illustrating the relationship between macrostructural brain properties (volume estimates of the overall cortex and white matter) and intelligence are depicted in the left column. Scatter plots illustrating the relationship between microstructural brain properties (isotropic diffusion averaged across the overall cortex and white matter) and intelligence are depicted in the right column. Results are based on partial correlation analyses with age and sex being used as controlling variables. Statistically significant partial correlation coefficients (N = 259, $p < .05$) are highlighted with black boxes.

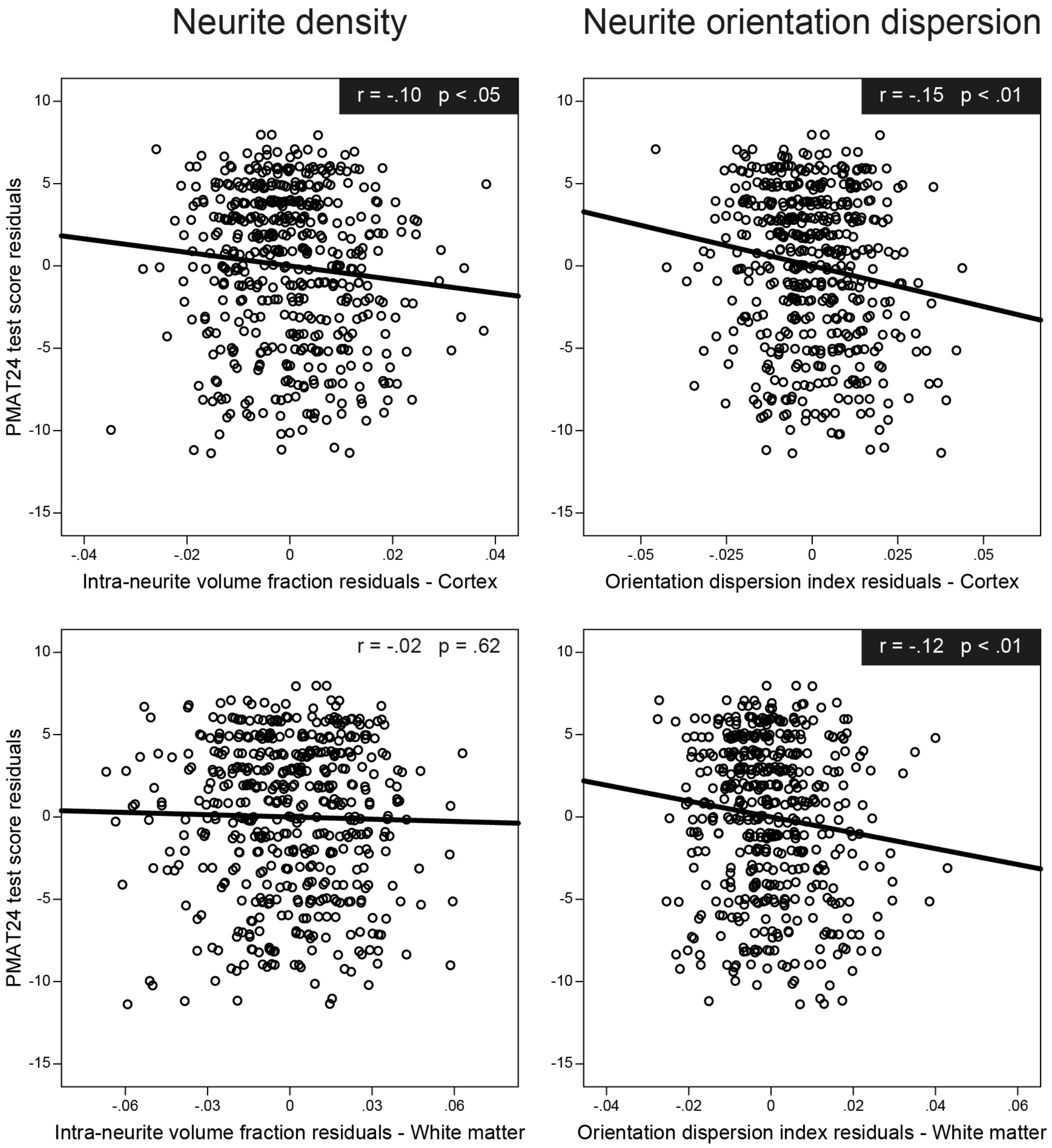

Supplementary Figure 2 - **Partial correlation analyses with data from sample S498 quantifying structure-function associations at the whole-brain level.** Scatter plots illustrating the relationship between neurite density and intelligence are depicted in the left column. Scatter plots illustrating the relationship between neurite orientation dispersion and intelligence are depicted in the right column. In all cases, microstructural measures were computed as mean values derived from the overall cortex (upper row) or white matter (lower row) respectively. Results are based on partial correlation analyses with age and sex being used as controlling variables. Statistically significant partial correlation coefficients (N = 498, $p < .05$) are highlighted with black boxes.

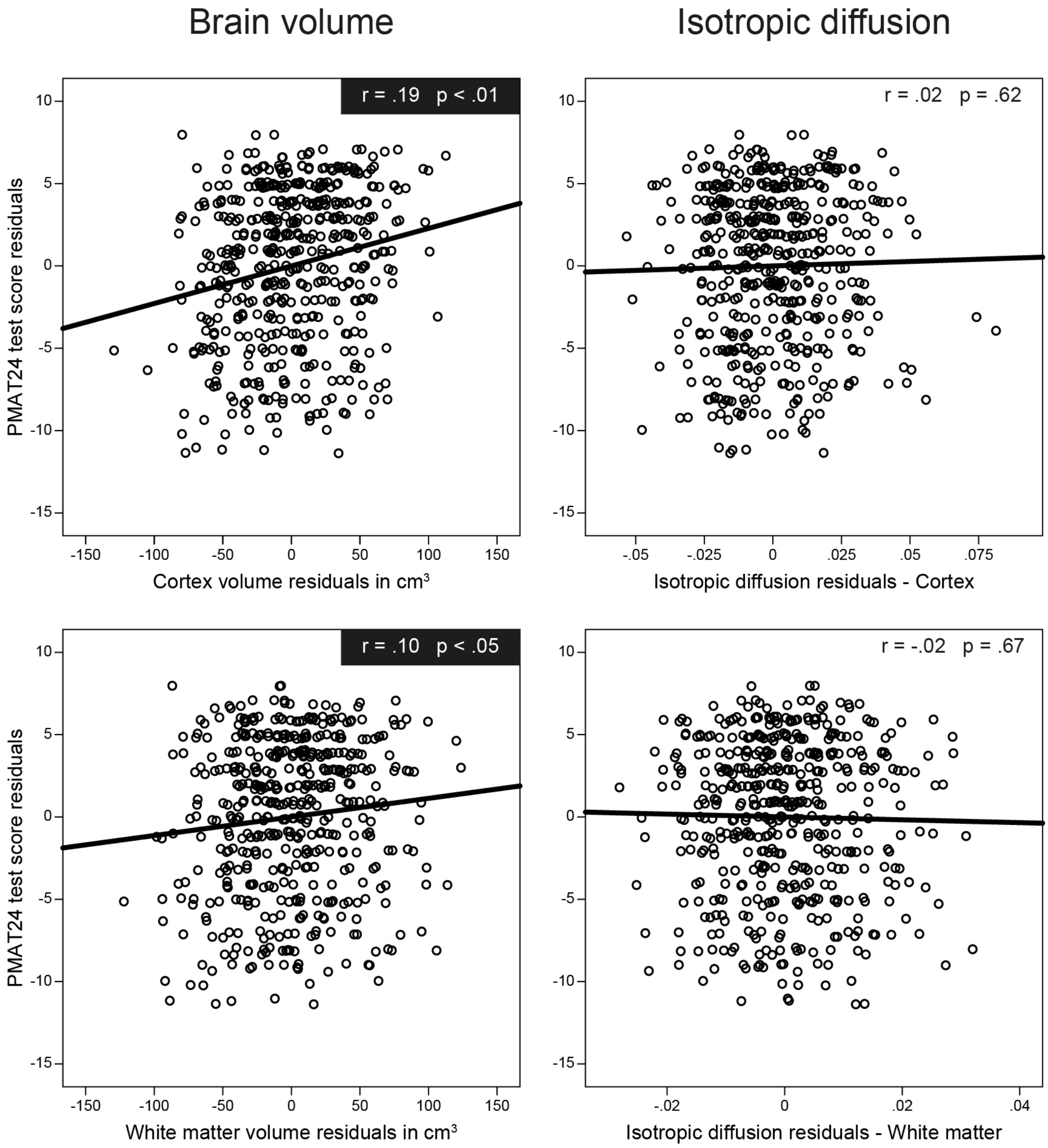

Supplementary Figure 3 - **Partial correlation analyses with data from sample S498 quantifying structure-function associations at the whole-brain level.** Scatter plots illustrating the relationship between macrostructural brain properties (volume estimates of the overall cortex and white matter) and intelligence are depicted in the left column. Scatter plots illustrating the relationship between microstructural brain properties (isotropic diffusion averaged across the overall cortex and white matter) and intelligence are depicted in the right column. Results are based on partial correlation analyses with age and sex being used as controlling variables. Statistically significant partial correlation coefficients ($N = 498$, $p < .05$) are highlighted with black boxes.

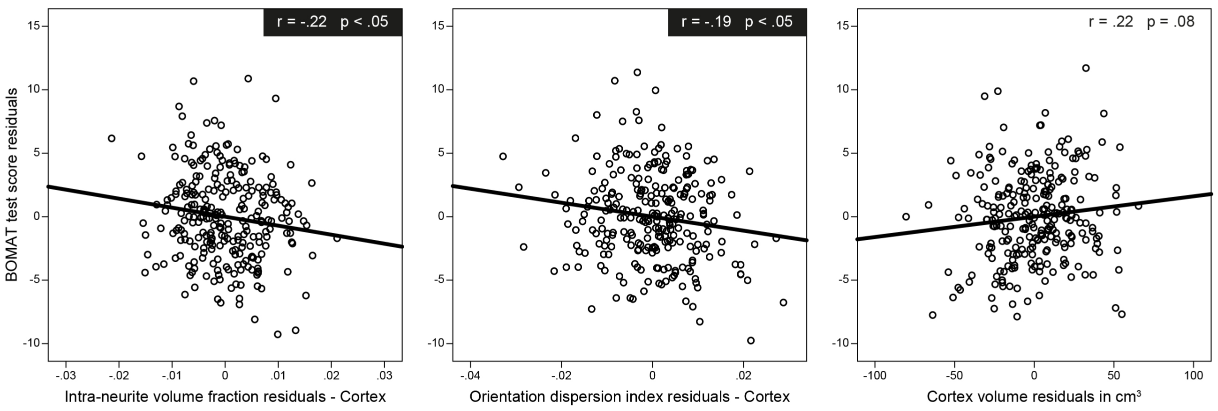

Supplementary Figure 4 - **Added variable plots with data from sample S259.** Added variable plots illustrating the unique contributions of $INVF_{Cortex}$ (neurite density in the cortex), ODI_{Cortex} (neurite orientation in the cortex) and VOL_{Cortex} (cortical volume) in predicting intelligence. Results are based on a multiple regression analysis with intelligence being regressed on age, sex, six microstructural brain properties ($INVF_{Cortex}$, $INVF_{WM}$, ODI_{Cortex}, ODI_{WM}, ISO_{Cortex}, ISO_{WM}) and two macrostructural brain properties (VOL_{Cortex}, VOL_{WM}). Statistically significant regression coefficients ($N = 259$, $p < .05$) are highlighted with black boxes.

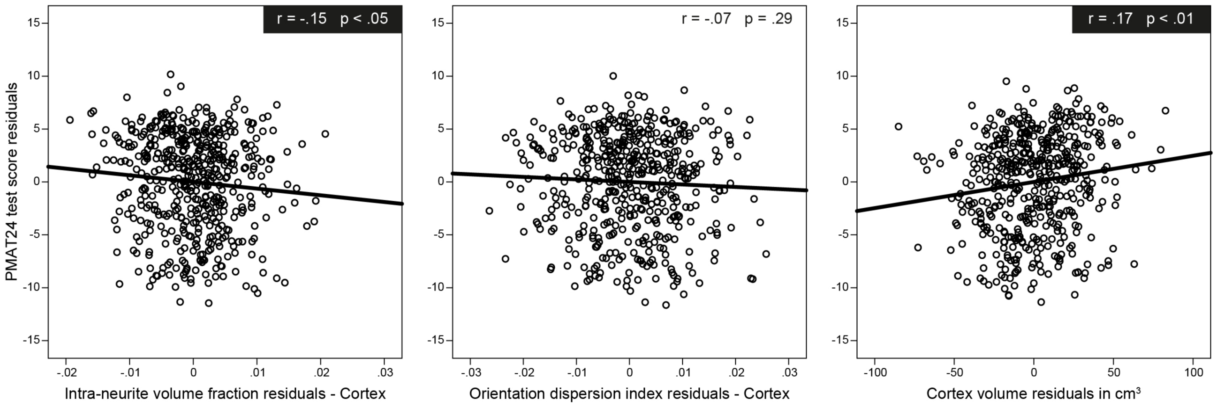

Supplementary Figure 5 - **Added variable plots with data from sample S498.** Added variable plots illustrating the unique contributions of $INVF_{Cortex}$ (neurite density in the cortex), ODI_{Cortex} (neurite orientation in the cortex) and VOL_{Cortex} (cortical volume) in predicting intelligence. Results are based on a multiple regression analysis with intelligence being regressed on age, sex, six microstructural brain properties ($INVF_{Cortex}$, $INVF_{WM}$, ODI_{Cortex}, ODI_{WM}, ISO_{Cortex}, ISO_{WM}) and two macrostructural brain properties (VOL_{Cortex}, VOL_{WM}). Statistically significant regression coefficients (N = 498, p < .05) are highlighted with black boxes.

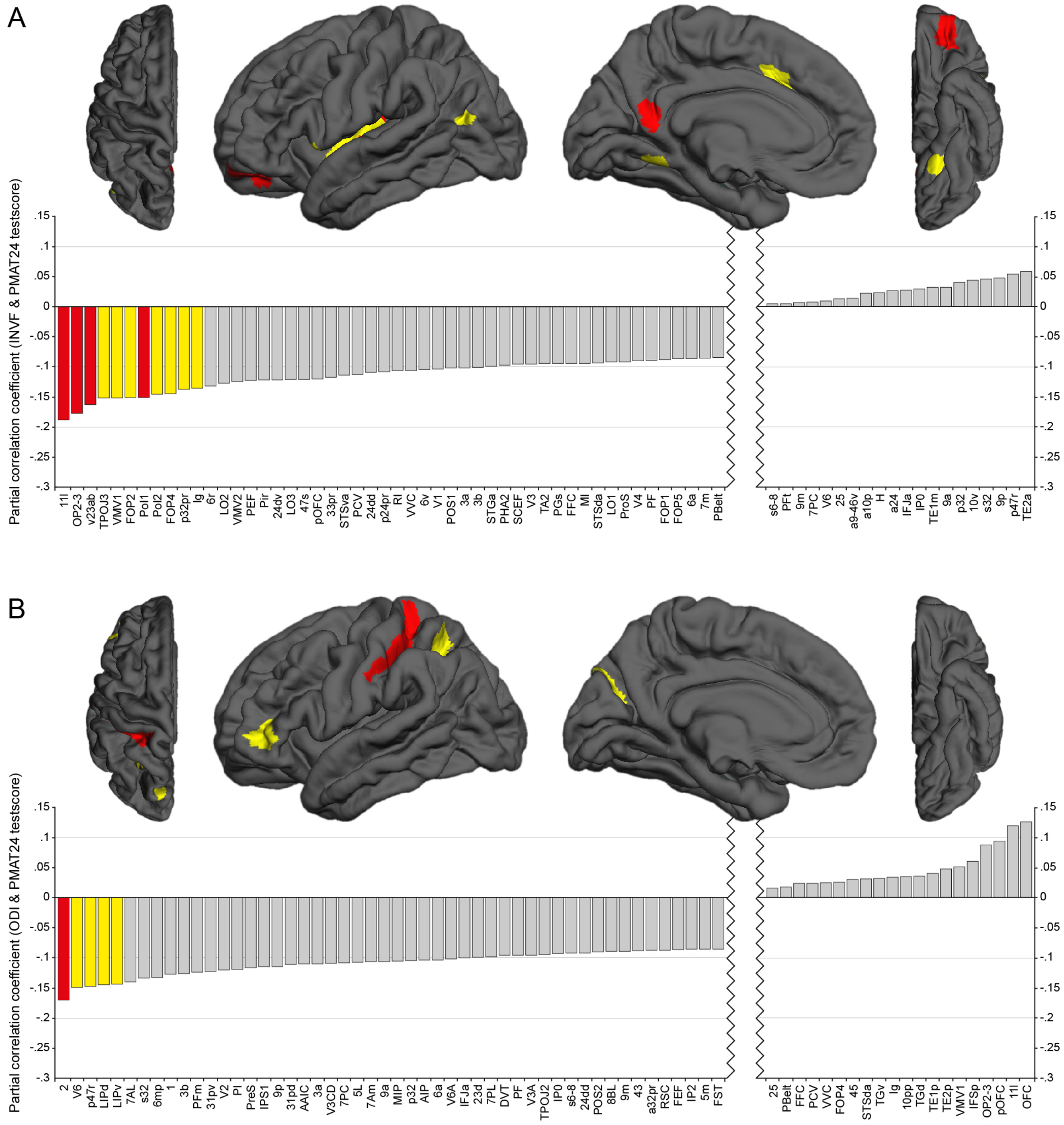

Supplementary Figure 6 - **Partial correlation analyses with data from sample S498 quantifying structure-function associations at the level of single brain regions.** For each hemisphere, 180 cortical brain regions were defined based on the multi-modal parcellation scheme provided by the Human Connectome Project. NODDI coefficients and volume measures from homotopic brain regions were averaged across both hemispheres, resulting in 180 mean values. Structure-function associations between $INVF_{Cortex}$ and intelligence (panel A) as well as ODI_{Cortex} and intelligence (panel B) were analyzed by means

of partial correlations with age, sex and the remaining cortical brain properties as controlling variables. FDR correction using the Benjamini-Hochberg method was applied to account for a total of 180 comparisons. Partial correlation coefficients are depicted as gray bars arranged by magnitude from negative to positive. Due to space restrictions, a middle portion of 110 brain regions exhibiting no significant structure-function associations is spared out. Statistically significant partial correlation coefficients that survived a critical FDR threshold of q = 0.05 (see Methods) are highlighted in either red or yellow. The yellow color marks significant partial correlation coefficients that are exhibited by brain regions from the P-FIT model of intelligence. Following this color scheme, respective brain regions are marked in either red or yellow on a cortical surface. $INVF_{Cortex}$ = intra-neurite volume fraction representing neurite density in the cortex, ODI_{Cortex} = orientation dispersion index of neurites in the cortex.

Supplementary Table 1. *Summary of correlation coefficients between variables from the S259 sample (N = 259)*

	$\mathbf{INVF_{Cortex}}$	$\mathbf{INVF_{WM}}$	$\mathbf{ODI_{Cortex}}$	$\mathbf{ODI_{WM}}$	$\mathbf{ISO_{Cortex}}$	$\mathbf{ISO_{WM}}$	$\mathbf{VOL_{Cortex}}$	$\mathbf{VOL_{WM}}$	**Age**
$\mathbf{INVF_{Cortex}}$	-	-	-	-	-	-	-	-	-
$\mathbf{INVF_{WM}}$	.60 (.00)**	-	-	-	-	-	-	-	-
$\mathbf{ODI_{Cortex}}$	.11 (.07)	.28 (.00)**	-	-	-	-	-	-	-
$\mathbf{ODI_{WM}}$	.22 (.00)**	.04 (.54)	.47 (.00)**	-	-	-	-	-	-
$\mathbf{ISO_{Cortex}}$	.40 (.00)**	.06 (.31)	.01 (.90)	.10 (.11)	-	-	-	-	-
$\mathbf{ISO_{WM}}$	.22 (.00)**	-.01 (.88)	-.10 (.10)	.10 (.10)	.71 (.00)**	-	-	-	-
$\mathbf{VOL_{Cortex}}$	-.12 (.06)	.01 (.90)	-.19 (.00)**	-.22 (.00)**	-.31 (.00)**	-.08 (.19)	-	-	-
$\mathbf{VOL_{WM}}$	.14 (.02)*	.11 (.08)	.07 (.30)	-.00 (.97)	-.09 (.16)	-.04 (.57)	.75 (.00)**	-	-
**Age**	.51 (.00)**	.19 (.00)**	.09 (.17)	.30 (.00)**	.35 (.00)**	.21 (.00)**	-.24 (.00)**	.06 (.32)	-

$INVF_{Cortex}$ = intra-neurite volume fraction representing neurite density in the cortex, $INVF_{WM}$ = intra-neurite volume fraction representing neurite density in the white matter, ODI_{Cortex} = orientation dispersion index of neurites in the cortex, ODI_{WM} = orientation dispersion index of neurites in the white matter, ISO_{Cortex} = isotropic diffusion in the cortex, ISO_{WM} = isotropic diffusion in the white matter VOL_{Cortex} = cortical volume, VOL_{WM} = white matter volume; p-values are given in brackets; * $p < .05$, ** $p < .01$

Supplementary Table 2. *Summary of correlation coefficients between variables from the S498 sample (N = 498)*

	**$INVF_{Cortex}$**	**$INVF_{WM}$**	**ODI_{Cortex}**	**ODI_{WM}**	**ISO_{Cortex}**	**ISO_{WM}**	**VOL_{Cortex}**	**VOL_{WM}**	**Age**
**$INVF_{Cortex}$**	-	-	-	-	-	-	-	-	-
**$INVF_{WM}$**	.60 (.00)**	-	-	-	-	-	-	-	-
**ODI_{Cortex}**	.59 (.00)**	.42 (.00)**	-	-	-	-	-	-	-
**ODI_{WM}**	.51 (.00)**	.07 (.11)	.65 (.00)**	-	-	-	-	-	-
**ISO_{Cortex}**	.53 (.00)**	.17 (.00)**	.28 (.00)**	.42 (.00)**	-	-	-	-	-
**ISO_{WM}**	.28 (.00)**	.17 (.00)**	.28 (.00)**	.37 (.00)**	.56 (.00)**	-	-	-	-
**VOL_{Cortex}**	-.03 (.57)	.12 (.01)**	-.03 (.50)	-.13 (.00)**	.03 (.52)	.27 (.00)**	-	-	-
**VOL_{WM}**	.09 (.04)*	.15 (.00)**	.17 (.00)**	.05 (.26)	.05 (.24)	.29 (.00)**	.82 (.00)**	-	-
**Age**	.26 (.00)**	.09 (.04)*	.22 (.00)**	.25 (.00)**	-.01 (.85)	-.12 (.01)**	-.12 (.01)*	.08 (.08)	-

$INVF_{Cortex}$ = intra-neurite volume fraction representing neurite density in the cortex, $INVF_{WM}$ = intra-neurite volume fraction representing neurite density in the white matter, ODI_{Cortex} = orientation dispersion index of neurites in the cortex, ODI_{WM} = orientation dispersion index of neurites in the white matter, ISO_{Cortex} = isotropic diffusion in the cortex, ISO_{WM} = isotropic diffusion in the white matter VOL_{Cortex} = cortical volume, VOL_{WM} = white matter volume; p-values are given in brackets; * p < .05, ** p < .01

Supplementary Table 3. *Summary of multiple regression analysis for variables predicting BOMAT test scores (N = 259, R^2 = .14)*

Variable	*B*	*SE B*	*β*
**$INVF_{Cortex}$**	-71.24	31.58	-.22*
**$INVF_{WM}$**	28.05	15.26	.15
**ODI_{Cortex}**	-54.77	22.49	-.19*
**ODI_{WM}**	9.18	33.66	.02
**ISO_{Cortex}**	-7.50	13.65	-.06
**ISO_{WM}**	41.84	32.99	.11
**VOL_{Cortex} in cm^3**	0.02	0.01	.22
**VOL_{WM} in cm^3**	0.00	0.01	-.05
**Age in years**	-0.02	0.06	-.03
**Sex**	0.06	0.57	.01
**Head motion**	0.64	1.49	.03

$INVF_{Cortex}$ = intra-neurite volume fraction representing neurite density in the cortex, $INVF_{WM}$ = intra-neurite volume fraction representing neurite density in the white matter, ODI_{Cortex} = orientation dispersion index of neurites in the cortex, ODI_{WM} = orientation dispersion index of neurites in the white matter, ISO_{Cortex} = isotropic diffusion in the cortex, ISO_{WM} = isotropic diffusion in the white matter VOL_{Cortex} = cortical volume, VOL_{WM} = white matter volume; Sex was represented as a dummy variable with males being labeled 0 and females 1; * $p < .05$, ** $p < .01$

Supplementary Table 4. *Summary of multiple regression analysis for variables predicting PMAT24 test scores (N = 498, R^2 = .08)*

Variable	*B*	*SE B*	*β*
**$INVF_{Cortex}$**	-63.10	31.98	-.15*
**$INVF_{WM}$**	15.64	12.69	.08
**ODI_{Cortex}**	-23.86	22.33	-.07
**ODI_{WM}**	-4.07	28.13	-.01
**ISO_{Cortex}**	40.71	15.36	.17**
**ISO_{WM}**	-30.55	25.45	-.07
**VOL_{Cortex} in cm^3**	0.02	0.01	.27**
**VOL_{WM} in cm^3**	-0.00	0.01	-.05
**Age in years**	0.08	0.07	.06
**Sex**	-0.13	0.58	-.01

$INVF_{Cortex}$ = intra-neurite volume fraction representing neurite density in the cortex, $INVF_{WM}$ = intra-neurite volume fraction representing neurite density in the white matter, ODI_{Cortex} = orientation dispersion index of neurites in the cortex, ODI_{WM} = orientation dispersion index of neurites in the white matter, ISO_{Cortex} = isotropic diffusion in the cortex, ISO_{WM} = isotropic diffusion in the white matter VOL_{Cortex} = cortical volume, VOL_{WM} = white matter volume; Sex was represented as a dummy variable with males being labeled 0 and females 1; * $p < .05$, ** $p < .01$
